# Management of Pregnancy during the COVID‐19 Pandemic

**DOI:** 10.1002/gch2.202000052

**Published:** 2020-10-22

**Authors:** Di Wu, Dong Fang, Renjie Wang, Dongrui Deng, Shujie Liao

**Affiliations:** ^1^ Department of Obstetrics and Gynecology Tongji Hospital Tongji Medical College Huazhong University of Science and Technology 1095 Jiefang Rd. Wuhan Hubei 430030 China; ^2^ Andrology Center and Department of Urology Peking University First Hospital Institute of Urology Peking University Beijing 100034 China

**Keywords:** coronavirus disease 2019, COVID‐19, obstetric management, pregnancy, Sars‐Cov‐2

## Abstract

Coronavirus disease 2019 (COVID‐19) is spreading worldwide. All aspects of pregnancy management from conception to delivery to puerperium as risks facing newborns are herein, reviewed. Maternal home management and prenatal care management protection, delivery timing or mode selection, delivery process management, and subsequent puerperal protection are crucial. In this Review, the features and treatment strategies, especially emphasizing the safety of antiviral drugs for pregnant women, the wearing of face masks, and practicing of personal hygiene (e.g., handwashing, disinfection, home cleaning, and ventilation) are reviewed as essential protective measures. It is recommended to provide online consultation, telemedicine, and remote fetal heart rate monitoring and set the flow point for prenatal examination to encourage prenatal examination at home or postponing examinations (except nuchal translucency at 11‐13+6 weeks, Oscar Test at 16 weeks, and fetal ultrasound at 20–24 weeks). It is shown that the precise formulation of follow‐up strategies for pregnant women with COVID‐19 is necessary.

## Graphical abstract

The 2019 novel coronavirus (2019‐nCoV) spread throughout the world rapidly. All aspects of pregnancy management from conception to delivery to puerperium, and including risks for newborns are reviewed in the paper.

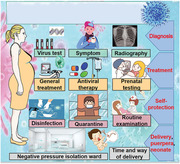

## 1 Introduction

Coronavirus disease 2019 (COVID‐19) is spreading worldwide, with at least 26 121 999 confirmed cases and 864 618 deaths until 4 September. Great efforts have been made to provide accurate diagnosis and effective treatment, and there have been many studies regarding the management of COVID‐19 patients.^[^ ^1^, ^2^, ^3^ ^]^

As a specific cohort, pregnant women with COVID‐19 require particular attention, since they were considered to be particularly susceptible to severe acute respiratory syndrome coronavirus 2 (SARS‐CoV‐2) because of their immunosuppressive state, change in hormonal levels, and physiological adaptive changes.^[^ ^4^, ^5^ ^]^ Until now, there have been several reports discussing the characteristics of outcomes of pregnant women and newborns; however, most have been limited by the small sample size and lack of data on the management of the whole gestation procedure, delivery, puerpera, and neonates.^[^ ^6^, ^7^ ^]^

In this narrative review, we collected relevant literature to illustrate recent developments on the features and treatment strategies in this cohort of patients and provide a recommended management for clinicians. We would also like to provide recommendations for management of pregnancy during the epidemic to decrease the possible risk of infection.

## 2 Diagnosis of COVID‐19 in Pregnant Women

The diagnosis of COVID‐19 should be based on symptoms, virus testing, and imaging. The following are recommended procedures for pregnant women suspect of COVID‐19:

### 2.1 Symptoms

Based on recent studies, the most common symptoms of pregnant women with COVID‐19 were fever and cough.^[^ ^8^ ^]^ Pregnant women with these symptoms or with a history of contact with suspected or confirmed cases should visit fever clinics to receive tests for COVID‐19.

### 2.2 Virus Test

The most commonly used diagnostic method is the real‐time reverse‐transcriptase polymerase chain reaction (RT‐PCR) assay on throat‐swab specimens.^[^ ^9^ ^]^ The reported sensitivity was 89%, the positive predictive value (PPV) ranged from 47.3% to 96.4%, and the negative predictive value (NPV) ranged from 96.8% to 99.9%.^[^ ^10^ ^]^ High‐throughput sequencing would be more accurate yet time‐consuming, and specific instruments are required.

Serum antibodies (IgG and IgM) of SARS‐CoV‐2 have drawn attention recently as a quick and accurate method for diagnosis of patients and asymptomatic carriers. Point‐of‐care lateral flow immunoassay can detect antibodies within 15 min.^[^ ^11^ ^]^ The sensitivities and specificities of serum IgM and IgG antibodies to diagnose COVID‐19 were 48.1% and 88.9%, and 100% and 90.9%, respectively.^[^ ^12^ ^]^ The presence of IgG means previous infection, or the patient might be recovered, and the presence of IgM means recent or current infection, while decreasing IgM and increasing IgG during repeated tests indicate that the patient has nearly recovered.^[^ ^13^ ^]^

### 2.3 Imaging Tests

All pregnant women suspected of COVID‐19 and those admitted to the hospital in an endemic area should receive low‐dose chest CT. CT can help identify suspected COVID‐19 patients even when RT‐PCR is negative.^[^ ^14^, ^15^ ^]^ The impact on the fetus of chest CT during pregnancy is very little.^[^ ^16^ ^]^ Multiple, patchy, ground‐glass opacity,^[^ ^4^ ^]^ crazy‐paving pattern, and consolidation shadows distributed in both lungs′ peripheral and subpleural areas are typical manifestation in pregnant women with COVID‐19.^[^ ^17^ ^]^ A meta‐analysis included 236 pregnant women with COVID‐19 demonstrated that 71% patients had positive CT finding.^[^ ^8^ ^]^ Chen et al. have found 88 of the 111 pregnant women in Wuhan (79%) who underwent chest CT had infiltrates in both lungs.^[^ ^18^ ^]^ Using RT‐PCR as a reference, the sensitivity, specificity, PPV, and NPV of chest CT in diagnosing COVID‐19 are 97%, 25%, 65%, and 83%, respectively.^[^ ^19^ ^]^ Also the study by our group has collected the information of 122 pregnant women with COVID‐19, and found 106 of the 122 pregnant women with positive CT findings (unpublished paper). These literatures could demonstrate that the use of chest CT was important in the diagnosis and evaluation of COVID‐19 in pregnant women. Another commonly reported laboratory finding is a normal or decreased count of white blood cells and lymphocytes.^[^ ^20^ ^]^

The criteria for suspected cases were as follows: history of exposure to SARS‐CoV‐2 infection 14 days before onset, with symptoms such as fever and cough, typical imaging features of coronavirus disease, and normal or low early leukocyte and lymphocyte counts. The confirmation was based on the confirmation of suspected cases, plus one of the following: positive RT‐PCR assay, high‐throughput sequencing, or the antibody test.^[^ ^21^ ^]^

## 3 Treatment of COVID‐19 in Pregnant Women

Pregnant women diagnosed with COVID‐19 should be admitted to the quarantine ward. Self‐care at home is not recommended, even though most reported cases were mild and had a good recovery.^[^ ^6^ ^]^ Until discharge criteria are met, pregnant women can be removed from isolation:1) body temperature is normal for at least three days and respiratory symptoms significantly improves; 2) there is evident absorption of acute exudative lesions on chest imaging; 3) results for two consecutive tests of the nucleic acid of SARS‐CoV‐2 (with an interval of at least one day) are negative;^[^ ^21^ ^]^ 4) no other obstetrical condition with vital signs.

### 3.1 General Treatment

General treatment was similar between pregnant women and general population. Patients should rest and receive adequate nutritional support. Monitoring of vital signs, symptoms (especially those related to respiratory and heart failure), FiO_2_ and complete blood count (CBC), liver and renal function, C‐reactive protein (CRP), and chest imaging are required. Oxygen therapy through nasal catheter or mask is essential.^[^ ^22^ ^]^ Some studies have shown that traditional Chinese herbs have been effective in treating mild COVID‐19 patients^[^ ^23^ ^]^ and could be considered for pregnant patients.

### 3.2 Antiviral Therapy

Owing to the near‐universal exclusion of drug trials in pregnant women, there was limited data of antiviral drugs in pregnant women. We have reviewed relevant publications and collected the information that might be beneficial to pregnant patients.

An international study of Remdesivir found that 68% of the participants improved, and 13% got worse and died after treatment.^[^ ^24^ ^]^ Several cases showed that remdesivir might be effective and safe for the treatment of severe COVID‐19 in pregnancy.^[^ ^25^, ^26^, ^27^ ^]^

Chloroquine has also been used for COVID‐19^[^ ^28^ ^]^ and has shown apparent efficacy in the treatment of COVID‐19.^[^ ^29^ ^]^ Some evidence indicated that chloroquine and hydroxychloroquine showed superior maternal and fetal safety and are worthy of consideration for pregnant women with COVID‐19.^[^ ^30^, ^31^ ^]^ However, high‐dose chloroquine's relevant side effect is systolic hypotension, which may exacerbate the hemodynamic changes from supine aortocaval compression by a gravid uterus.^[^ ^32^ ^]^

A number of antiviral drugs have been demonstrated to be safe and effective in pregnancy. During H1N1 pandemic influenza infection, studies showed that clinically significant oseltamivir doses in pregnant women have no potential to affect fetal development adversely.^[^ ^33^ ^]^ In October 2018, the American College of Obstetricians and Gynecologists (ACOG) recommends that pregnant women with suspected or confirmed influenza could receive antiviral therapy (oseltamivir or zanamivir) based on current situation.^[^ ^34^ ^]^ These agents are potentially beneficial and further clinical studies are required.

But with limited data, pregnant women should be cautious to use these agents. Clinicians should monitor patients for adverse effects and the potential toxicity to decrease the risk of sudden cardiac death or other complications.

### 3.3 Treatment for Severe Cases

The intensive care unit (ICU) should be considered in patients with abnormal vital signs, presence of shock or other organ failure, progressively decreased peripheral lymphopenia, and inflammatory factors (e.g., interleukin‐6 (IL‐6), C‐reactive protein, or lactate), or rapidly progressed pulmonary lesions in a short time. Mechanical ventilation is used for patients with respiratory failure or low FiO_2_.^[^ ^21^ ^]^ Continuous renal replacement therapy (CRRT), hemopurification, or other high‐level supportive therapy can be considered when required. These treatments have been proved to be effective in severe pregnant patients.^[^ ^4^, ^35^, ^36^ ^]^

### 3.4 Special Treatment for Pregnant Women According to Gestational Age

For patients within 28 weeks of gestation, the main treatment was supportive and antiviral treatment. Gestation weeks should be prolonged as much as possible, if the maternal and fetal conditions are stable. For those with gestational age over 28 weeks, the condition of the fetus should be closely monitored, and dexamethasone should be used for maturation. Pregnancy can be terminated when the infection tends to be out of control or with obstetrical indication.^[^ ^37^ ^]^

### 3.5 Prophylaxis: Persons at Risk of Infection with COVID‐19

Any drug currently administered before or after exposure is not effective in preventing infection with SARS‐CoV‐2, as demonstrated in clinical trials.^[^ ^38^ ^]^ Currently, there no approved vaccines for the prevention of COVID‐19 in pregnant women, owing to the limitation of clinical trials. More clinical trials are needed for the utilization of vaccine of COVID‐19 in pregnant women.

## 4 Pregnancy Management during COVID‐19 Pandemic

Absolute quarantine is difficult for pregnant women because care from family members and routine examinations in hospitals are required. In this section, we discuss how to prevent pregnant women from the infection of SARS‐CoV‐2 during this pandemic.

### 4.1 Self‐Protection in Daily Life

During the pandemic era, pregnant women should be asked to stay at home, except for medical reasons, especially in endemic areas. Strict quarantine is required when suspected or confirmed cases are present in family members.^[^ ^39^ ^]^ A convenient and effective method for prevention of infection is wearing face masks.^[^ ^40^ ^]^ Surgical masks can significantly decrease the expiration of SARS‐CoV‐2 (or flu),^[^ ^41^ ^]^ and N95 or medical masks can block at least 90% of the virus in aerosols.^[^ ^42^ ^]^ In addition, personal hygiene (e.g., handwashing, disinfection, home cleaning, and ventilation) is important.^[^ ^39^ ^]^ Studies have shown that SARS‐CoV‐2 can stay in air or on surfaces for up to nine days. Also, 62–71% alcohol, 0.5% hydrogen peroxide, or 0.1% sodium hypochlorite can kill SARS‐CoV‐2 within a minute.^[^ ^43^ ^]^ Therefore, we put forward the following practical suggestions: spray or wipe with 500 mg L^−1^ chlorine‐containing disinfectant on high contact surfaces (such as tables, hard‐backed chair, doorknob, electric lamp switch, telephone, tablet computer, desk, and toilet) daily and wipe with water after acting for 30 min to prevent corrosion; wash hands with 60% alcohol hand sanitizer for at least 20 s before and after eating, sneezing or contacting public goods; and use disposable bowls and chopsticks or boil for 15 min before and after using dishes and chopsticks.

### 4.2 Self‐Protection in Hospitals

There is a risk of infection during routine examinations in clinics. Previously, we studied 48 pregnant women infected with COVID‐9, 27 (56.25%) of whom were infected because of obstetric examination (unpublished data). During the pandemic, routine pregnancy examination should be performed in consideration of the gestational weeks, importance of the examinations, and individual condition of each pregnant woman.^[^ ^44^ ^]^ The following examinations should not be omitted during pregnancy: nuchal translucency (NT) at 11‐13+6 weeks, Oscar Test at 15–20 weeks, and fetus ultrasound at 20–24 weeks. Routine examinations at other weeks can be postponed or canceled under the judgment of clinicians if the pregnant woman has no fever or respiratory symptoms, no individual comorbidities or obstetrical complications, and no abnormal conditions such as vaginal bleeding or abdominal pain. It is necessary to provide online consultation, telemedicine, and remote fetal heart rate monitoring, and set the flow point for prenatal examination to encourage prenatal examination at home and decrease the frequency of examinations in the hospital.

Reservation is required before visiting clinics to avoid crowding. Other vital suggestions include wearing face masks, avoiding public transportation, decreasing contact with other persons, and washing of hands regularly.^[^ ^44^ ^]^ Over anxiety should be avoided in visiting hospitals with obstetric symptoms.^[^ ^45^ ^]^

## 5 Management of Delivery in Pregnant Women with COVID‐19

### 5.1 Time of Delivery

Infected by SARS‐CoV‐2 is not an absolute indicator of immediate termination of pregnancy.^[^ ^6^, ^7^ ^]^ In those between 28 and 34 weeks, expected management can be carried out if the patient with pneumonia is stable or under control and no abnormality in fetus monitoring or other complications. Maturation by dexamethasone can be considered during this period. When gestation week is over 34, a timely termination of pregnancy can be considered because of the high possibility of newborn survival. However, there are some reports of full‐term delivery after expectation, and there is no evidence of a higher risk of fetal infection or other abnormalities during pregnancy.^[^ ^46^ ^]^ There was also a reported case that a healthy baby was delivered 8 weeks after his mother's recovery of COVID‐19.^[^ ^47^ ^]^ The study by our group has followed up five pregnant women with COVID‐19 in convalescence (1–4 months after recovery), all of them have given birth to babies without infection of COVID‐19 or other diseases (unpublished paper). However, until now most current studies are cross‐sectional study while long‐term cohort studies are lacking. We need to follow up the pregnant women recovered from COVID‐19 in the near future.

Severe and critical conditions signal the importance of timely termination of pregnancy, including respiratory distress (RR ≥ 30 beats per min) or at rest, oxygen saturation ≤93% or arterial partial pressure of oxygen (PaO2)/oxygen concentration (FiO_2_) ≤300 mmHg, mechanical ventilation required, shock, or other organ failure requiring ICU.^[^ ^21^ ^]^ In addition, timely termination of pregnancy is required when obstetric termination of pregnancy occurs.

### 5.2 Way of Delivery

Cesarean section (CS) should be considered with obstetric indications, including fetal distress, placenta abruption, cephalopelvic disproportion, scarred uterus, abnormal fetal position, placenta praevia, twins or multiplets, prolapse of umbilical cord, pregnant women with severe comorbidities and complications, macrosomia, the pregnant woman asked for a cesarean section, genital tract malformation and infection, and pregnancy complicated with tumor.^[^ ^48^ ^]^ The threshold for CS should be flexible for pregnant women with COVID‐19, such as the threshold regarding the delay in the first stage of labor.^[^ ^49^ ^]^ However, CS is not the only choice. When there is no contradiction for vaginal delivery and the COVID‐19 is mild or moderate, vaginal delivery can be considered under strict monitoring. Patients with severe pneumonia or evidence that might be worse during vaginal delivery should receive CS.

### 5.3 Protection during Delivery

Delivery should be performed in negative pressure isolation ward.^[^ ^50^ ^]^ Doctors and nurses need to be fully protected during the entire delivery procedure, equipped with N95 face masks, protective clothing, goggles. Intraspinal anesthesia (preferred) or general anesthesia with endotracheal intubation (for severe or critically ill patients) can be adopted for delivery.^[^ ^51^ ^]^ General and epidural anesthesia can be adopted for CS in pregnant women with COVID‐19, while a study found a higher risk of hypotension under epidural anesthesia.^[^ ^52^ ^]^ General anesthesia was also regarded as having the risk of virus dissemination, deterioration of disease, or the impact on the fetus by a review.^[^ ^49^ ^]^ Before the induction of anesthesia, continuous high‐flow oxygen should be administered with a mask, and rapid induction of anesthesia should be adopted to avoid choking cough. After the patient's consciousness disappears, a double‐layer saline‐moist gauze should be placed in the mouth and nose to start low‐tidal volume high‐frequency ventilation to avoid lung injury and virus dispersion caused by increased airway pressure. During surgery, physicians should protect the patient's blood, secretions, amniotic fluid, excretions, and the aerosols generated when using surgical equipment. After surgery, all articles in the operating room should be disinfected, and the specimens should undergo pathological examination according to biosafety procedures. A few rules need to be noted: Health‐care workers who collect specimens should use appropriate personal protective equipment (PPE) and should be trained in safe handling practices; specimens for transport should be in leak‐proof specimen bags; and procedures with the potential to generate aerosols or droplets should be handled in a certified Class II Biological Safety Cabinet (BSC).^[^ ^53^ ^]^

## 6 Management of Puerpera

Pregnant women become more susceptible to infection after delivery. The severity might deteriorate, and asymptomatic SARS‐CoV‐2 carriers might exhibit symptoms; therefore, critical care is required. Puerpera should be transferred back to the isolated area; and vital signs, uterine contraction, vaginal bleeding, and wound healing should be monitored. Effects to increase uterine contraction should be made, and supportive and antiviral treatment should be continued. Breastfeeding is not recommended for uncured COVID‐19 patients.^[^ ^54^ ^]^ For breastfeeding, the United States Centers for Disease Control and Prevention (CDC) provides guidance to pump breast milk out with a dedicated pump and feed it by healthy individuals. Puerpera can be discharged from the hospital or released from quarantine when 1) body temperature is normal for at least three days and respiratory symptoms significantly improves; 2) there is evident absorption of acute exudative lesions on chest imaging; 3) results for two consecutive tests of the nucleic acid of SARS‐CoV‐2 (with an interval of at least one day) are negative; 4) the abdominal incision/perineum incision is healed well, vaginal bleeding is limited and uterus contraction is satisfactory.^[^ ^21^ ^]^

## 7 Management of Neonates Born to COVID‐19 Patients

There have been reports of newborns with positive antibodies to SARS‐CoV‐2^[^ ^55^ ^]^ and one case of positive nuclei acid test in newborns 30 h after birth.^[^ ^56^ ^]^ However, no conclusion has drawn regarding whether the antibodies were transmitted from the mothers or intrauterine infection occurred.^[^ ^57^ ^]^ It is necessary to reduce the risk of transmission during and after delivery. The umbilical cord should be cut as early as possible without compression or delayed ligation. The oral and nasal secretions of the newborn should be cleaned, and the body should be dried as first as possible to avoid the infection through maternal peripheral blood or amniotic fluid.

Some neonates with COVID‐19 had almost no symptoms, and it was difficult to judge the changes in white blood cells and lymphocytes because of the various reference ranges. In terms of the SARS‐CoV‐2 RNA tests in throat and anal swab samples, there may have been sampling errors and false negative or false positive results. The antibody of SARS‐CoV‐2 may become a powerful method for identifying the infections and the chest CT images appear to be the most important for diagnosis and prognosis of COVID‐19 among neonates.^[^ ^58^ ^]^

Positive specific IgM antibodies usually appear at 3–5 days of COVID‐19 onset. Positive IgM antibody indicates recent infection, and positive IgG antibody indicates previous infection.^[^ ^21^ ^]^ The rise of IgM is generally considered to be an indicator of intrauterine or perinatal infection. If the newborn has positive IgM and IgG, he/she should be hospitalized into isolated wards and received the nucleic acid of the throat sample, CT imaging, and treatments including antivirus drugs, oxygen, and nutrition supplies. When required, he/she should be hospitalized in the intensive care unit.

## 8 Management of Pregnant Women with COVID‐19 during the Rehabilitation Period

### 8.1 Common Recommendations

Positive RT‐PCR retests and significant lesions that were not absorbed in discharged COVID‐19 patients in some studies indicated that discharged isolation COVID‐19 patients need strict follow‐up with both nucleic acid testing, CT examination, and antibody testing to reduce the insidious risk of persistent infection.^[^ ^59^ ^]^ It is recommended that all discharged COVID‐19 pregnant women are transferred to a designated medical unit for extra 14 days of quarantine and observation. We examined the levels of immune cells and immune factors in patients (eight members of the familial cluster) with COVID‐19 during the rehabilitation period, and we found that total T cell count, cytotoxic T lymphocyte count, and T helper cell count in four members were relatively lower than the reference range, which was somewhat similar to SARS patients.^[^ ^60^ ^]^ Thus, more attention should be paid to protect them from other infections.

### 8.2 Special Precautions

In addition to the above common recommendations for pregnant women after discharge, there are special precautions for pregnant women at different stages (with COVID‐19). First, after discharge in the first and second trimesters, the pregnant woman needs to strengthen nutrition and exercise. Common complications inspection (as mentioned above) is necessary to monitor the growth and development of the fetus and malformation at the same time. Second, pregnant women in the rehabilitation period (with COVID‐19) in the third trimester pregnancy need to undergo a basic review of antibodies and CT before delivery. IgM (+) and significant lesions not absorbed may signal that the virus has not been completely cleared. Delivery then should be performed according to the above‐mentioned recommendations for current COVID‐19 patients. Finally, since the immune system of pregnant women in the rehabilitation period (with COVID‐19) may not recover immediately, the most critical measure during puerperium is to improve immunity and protect against postpartum infection.

## 9 Conclusion

Practical methods are required to prevent pregnant women from being infected by SARS‐CoV‐2 and help in early diagnosis and treatment. Without clear evidence of intrauterine vertical transmission, the management of pregnant women with COVID‐19 should consider obstetrical conditions, fetal development, and the severity of COVID‐19. All aspects of management, from preconception to delivery to puerperium, as well as newborns, need to be refined. Pregnancy management is the cornerstone of overcoming the epidemic, and delivery is a crucial trigger. Maternal home management and prenatal care management protection, delivery timing, mode selection, delivery process management, and subsequent puerperal protection are essential to obtain the final healthy newborn. Fortunately, most patients have good recovery, and full‐term vaginal delivery could be expected.

## Data Availability

WHO coronavirus disease (COVID‐2019) situation reports are available at https://www.who.int/emergencies/diseases/novel‐coronavirus‐2019/situation‐reports/. NIH COVID‐19 treatment guidelines are available at https://covid19treatmentguidelines.nih.gov/introduction/. Resources associated with coronavirus disease 2019 are available at https://www.cdc.gov/coronavirus/2019‐nCoV/lab/lab‐biosafety‐guidelines.html.

## Conflict of Interest

The authors declare no conflict of interest.

## Author Contributions

D.W., D.F., and R.W. contributed equally to this work. All authors were involved in the writing and revision of the manuscript. All authors read and approved the final version.
